# Transcriptome analysis reveals core lncRNA-mRNA networks regulating melanization and biomineralization in *Patinopecten yessoensis* shell-infested by *Polydora*

**DOI:** 10.1186/s12864-023-09837-w

**Published:** 2023-11-29

**Authors:** Yiying Wang, Junxia Mao, Zhiyue Fan, Yunna Hang, AnQi Tang, Ying Tian, Xubo Wang, Zhenlin Hao, Bing Han, Jun Ding, Yaqing Chang

**Affiliations:** Key Laboratory of Mariculture & Stock Enhancement in North China’s Sea, Ministry of Agriculture and Rural Affairs, Dalian Ocean University, Dalian, China

**Keywords:** *Patinopecten yessoensis*, *Polydora*, LncRNAs, Melanization, Biomineralization

## Abstract

**Background:**

*Patinopecten yessoensis*, a large and old molluscan group, has been one of the most important aquaculture shellfish in Asian countries because of its high economic value. However, the aquaculture of the species has recently been seriously affected by the frequent outbreaks of *Polydora* disease, causing great economic losses. Long non-coding RNAs (lncRNAs) exhibit exhibit crucial effects on diverse biological processes, but still remain poorly studied in scallops, limiting our understanding of the molecular regulatory mechanism of *P. yessoensis* in response to *Polydora* infestation.

**Results:**

In this study, a high-throughput transcriptome analysis was conducted in the mantles of healthy and *Polydora*-infected *P. yessoensis* by RNA sequencing. A total of 19,133 lncRNAs with 2,203 known and 16,930 novel were identified. The genomic characterizations of lncRNAs showed shorter sequence and open reading frame (ORF) length, fewer number of exons and lower expression levels in comparison with mRNAs. There were separately 2280 and 1636 differentially expressed mRNAs and lncRNAs (DEGs and DELs) detected in diseased individuals. The target genes of DELs were determined by both co-location and co-expression analyses. Functional enrichment analysis revealed that DEGs involved in melanization and biomineralization were significantly upregulated; further, obviously increased melanin granules were observed in epithelial cells of the edge mantle in diseased scallops by histological and TEM study, indicating the crucial role of melanizaiton and biomineralization in *P. yessoensis* to resist against *Polydora* infestation. Moreover, many key genes, such as *Tyrs*, *Frizzled*, *Wnts*, *calmodulins*, *Pifs*, *perlucin*, *laccase*, *shell matrix protein*, *mucins* and *chitins*, were targeted by DELs. Finally, a core lncRNA-mRNA interactive network involved in melanization and biomineralization was constructed and validated by qRT-PCR.

**Conclusions:**

This work provides valuable resources for studies of lncRNAs in scallops, and adds a new insight into the molecular regulatory mechanisms of *P. yessoensis* defending against *Polydora* infestation, which will contribute to *Polydora* disease control and breeding of disease-resistant varieties in molluscs.

**Supplementary Information:**

The online version contains supplementary material available at 10.1186/s12864-023-09837-w.

## Background

**Fig. 1:**
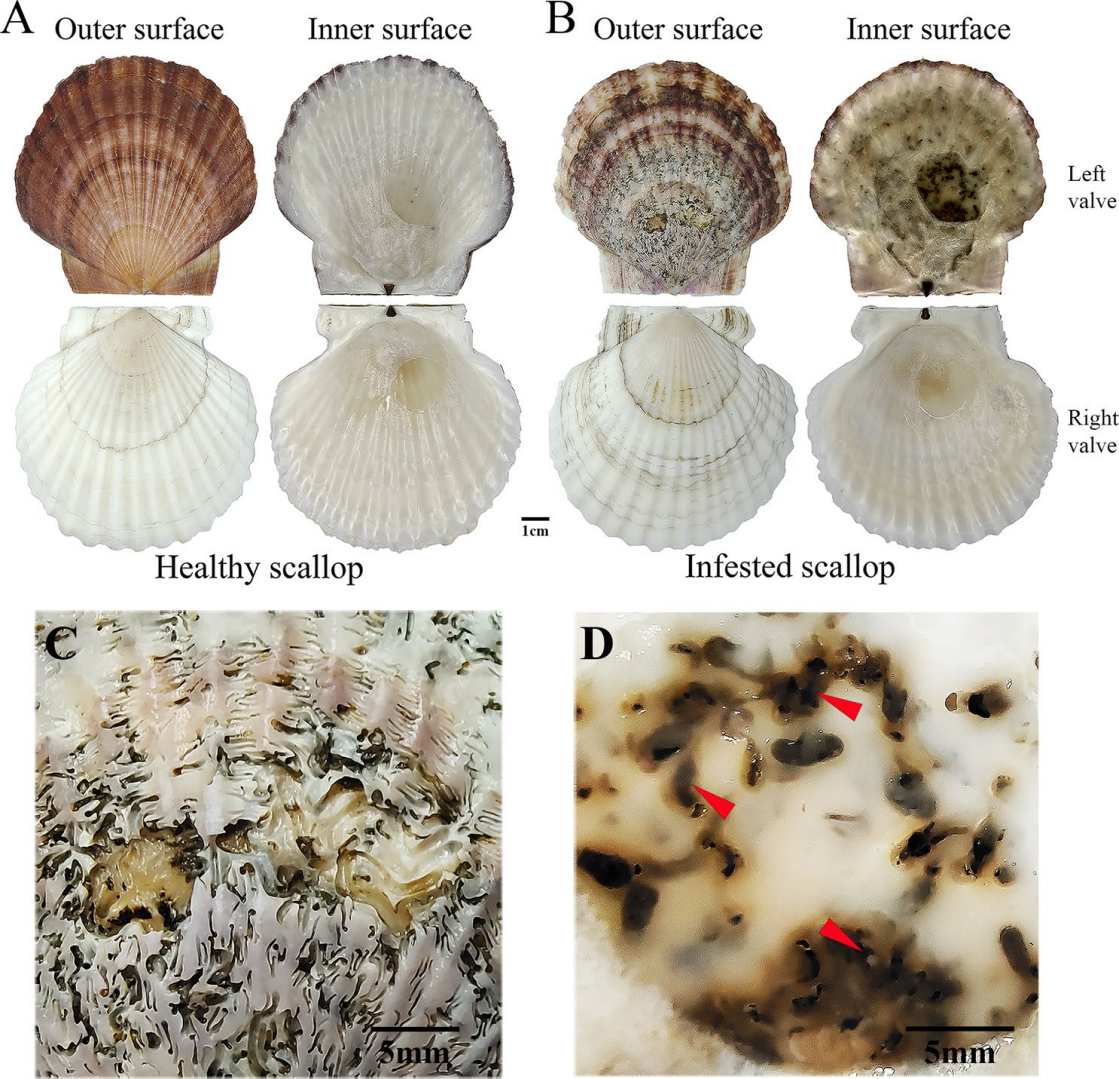
Shells of healthy and *Polydora*-infected *P. yessoensis*. Shell outside and inside of healthy (**A**) and *Polydora*-infected (**B**) *P. yessoensis*. (**C**) Enlarged view of the outside of damaged shell excavated by *Polydora*. (**D**) Enlarged view of mineralized and melanistic protuberances formed in the inside of infested shell. Red arrows indicate the mineralized and melanistic protuberances

*Patinopecten yessoensis* (Yesso scallop), a large and ancient molluscan species, is naturally distributed along the coastlines of northern Korea, northern Japan, and the Russian Far East [1]. *P. yessoensis* has been one of the most important aquaculture shellfish in Asian countries because of its high economic value. However, the growth, quality and even the survival of the species has recently been seriously threatened by the frequent outbreaks of *Polydora* infestation, leading to a large economic loss [2–4]. *Polydora*, one kind of spionid worms, mainly parasitize in the left valves of *P. yessoensis* through excavating tunnels, which seriously damages shell structures and makes shells particularly fragile [5–7]. When the infection extremely severe, the shell will be drilled through, bringing the scallop soft body directly exposed to diverse pathogens in the environment [8]. Consequently, numerous mineralized and melanistic protuberances are formed in the inner side of shells to resist the invasion (as showed in Fig. 1). Therefore, melanization and biomineralization probably play vital roles for *P. yessoensis* to defend against the *Polydora* infestation, but the underlying regulatory mechanism is still unclear.

The calcified shells are considered one major evolutionary innovation contributing to the success of molluscs, and provide a critical physical line to protect the species from predation, pathogens and parasites infection, and environmental stresses [9, 10]. The shells usually occur in fabulous and diverse colors, which has been widely recognized and appreciated [11]. The mechanisms of shell biomineralization and pigmentation are always two hot research issues in molluscs [12]. Melanization is a common bioprocess in molluscan shell coloration. In invertebrates, it is also a significant mechanism for foreign objects encapsulation and wound healing [13]. Melanization is sometimes accompanied with biomineralization that melanized invaders will be embedded in a new-formed calcified shell layer, as found in brown ring disease (BRD) of clams and *Roseovarius* oyster disease (ROD) of oysters [13–16]. Though many studies have been conducted to identify genes or pathways involved in shell biomineralization and pigmentation [12, 17–21], the molecular mechanisms of which have still not been thoroughly elucidated due to their high complexity.

Long non-coding RNAs (lncRNAs), occupying the vast majority of non-coding RNAs (ncRNAs), are those RNA transcripts with the length longer than 200 nucleotides, and structurally resemble mRNAs, but have low abilities to code functional proteins [22, 23]. LncRNAs generally show poor sequence conservation, and high tissue and spatiotemporal expression specificity [24, 25]. LncRNAs have been revealed involved in gene expression regulation transcriptionally or post-transcriptionally [26], but their functional mechanisms are still needed fully characterized. Studies suggest that lncRNAs regulate expressions of genes separately in adjacent or distant genomic regions, i.e., *cis*- or *trans*-acting [27, 28].

LncRNAs exhibit crucial effects on diverse biological processes in vertebrates, such as reproduction, development, metabolism, immunity, sex determination and differentiation and so on [28–33]. With the prevalence of the next-generation sequencing, characterizations and expression patterns of lncRNAs in molluscs have been gradually investigated, and they are found functioning in diverse bioprocesses, covering immune and stress response [34–38], muscle growth [39], gametogenesis [40] and larval development [41]. Emerging evidence has also revealed that lncRNAs are involved in the modulation of shell biomineralization and pigmentation in molluscs [42–46]. For example, chorions peroxidase and its *cis*-acting lncRNA TCONS_00951105 were predicted to exhibit a key function in melanin biosynthesis in Pacific oysters (*Crassostrea gigas*) by RNA-seq [42]; in manila clam (*Ruditapes philippinarum*), 14 differential expressed mRNAs and their interactive 77 lncRNAs were thought to be involved in the pathways of melanin synthesis and porphyrin metabolic [45]; besides, a lncRNA named LncMSEN2 were identified functioning in exoskeleton formation in the pearl oyster (*Pinctada fucuta martensii*) [47]. However, most reports focused on species of oysters, clams and abalones, especially oysters, and almost no relevant research has been conducted in scallops, limiting our comprehensive understanding of the regulatory mechanisms of lncRNAs in molluscs.

In this work, a genome-wide of lncRNAs in the mantle tissues of healthy and *Polydora*-infected *P. yessoensis* was first investigated by RNA sequencing. The primary genomic features and expression patterns of the lncRNAs were systematically analysed. Differentially expressed genes and lncRNAs induced by *Polydora* infestation were identified. Integrative expressions of lncRNA-mRNA were examined and functions of differentially expressed target genes were further elucidated. Finally, a core lncRNA-mRNA network regulating melanization and biomineralization were constructed and validated. This work provides valuable resources for the study of lncRNAs in scallops, and adds a new insight into molecular regulatory mechanisms of *P. yessoensis* responding to *Polydora* infestation.

## Materials and methods

### Sample collection

*Polydora* infected and healthy *P. yessoensis* at two years old were collected from Dalian Zhangzidao sea area (Liaoning, China). All the samples were then cultured under lab conditions with aerated and filtered seawater at the temperature of about 10 ℃. The scallops were feed with the algae *Chlorella* sp. twice a day, and the seawater were changed once a day. After one-week acclimation, the scallops were dissected, and the left mantle tissues were sampled. For the diseased group, those badly infected scallops with most area of the left valves damaged were chosen for the following experiments (Fig. 1). For the experiments of RNA sequencing and quantitative real-time PCR (qRT-PCR), the tissues were instantly frozen in liquid nitrogen and then stored at a -80 ℃ freezer. For histological and ultrastructural observation, the tissues were separately fixed in Bouin’s solution and TEM (transmission electron microscope) fixative (JIJIA Biotechnology, China).

### Histological procedures

For histological study, the mantle tissues were fixed in Bouin’s solution for 24–72 h. Then, specimens were dehydrated in a graded ethanol series and next embedded in paraffin. A Leica RM2255 microtome (Leica, Germany) was used to produce serial sections with the thickness of 4 μm. Followingly, the sections were dewaxed and stained with hematoxylin and eosin (HE). Finally, the sections were observed with a Leica DM4-B microscope equipped with a Leica DM6200 camera (Leica, Germany) for digital images capture.

### Transmission electron microscopy

For transmission electron microscopy, after 2–4 h fixation and immersion in phosphoric acid buffer, the specimens were post-fixed in 1.0% OsO_4_ solution (pH 7.4) for 2 h. A graded ethanol series were used to dehydrate specimens, which were then embedded in 812 epoxy resin (SPI, China). Next, a Leica UC7 microtome (Leica, Germany) was used to produce ultrathin Sects. (60–80 nm). Afterward, the sections were contrasted with uranyl acetate and lead citrate. Finally, the sections were observed on a JEM1400PLUS electron microscope (Japan).

### RNA isolation, library construction and sequencing

Total RNAs of every individual were extracted by using the RNAprep pure tissue kit (Tiangen, China) following the instruction. The integrity of the RNA was estimated by 1% agarose gel electrophoresis. The purity and concentration were detected by a Bioanalyzer 2100 system (Agilent Technologies, USA). A NEBNext Ultra Directional RNA Library Prep Kit for Illumina (NEB, USA) was used for RNA-seq libraries construction with high-quality RNA, following the instruction. Briefly, rRNA (ribosomal RNA) was first removed from total RNAs, and then the remaining RNAs were fragmented. Followingly, the first cDNA strand was produced with reverse transcriptase and random hexamer primers. Then, the second cDNA strand was synthesized with RNase H and DNA polymerase I. After terminal repair, 3’ terminal adenosylation and adaptor ligation, cDNA fragments with 250–300 bp were selected for polymerase chain reaction (PCR). Three individuals separately for healthy and infested groups were selected as biological replicates for library construction. Finally, a total of six sequencing libraries were constructed, and subjected to 150 bp paired-end sequencing with the Illumina NovaSeq 6000 platform.

### Data processing

Raw sequencing data were processed with the software fastp [48] to remove adapters and low-quality reads, which contained unidentified nucleotides (N) > 10% or > 50% bases of a read with quality value ≤ 5. The software Hisat2 v2.0.5 [49] was used to map the high-quality clean reads to the genome of *P. yessoensis* [50] with the main parameters of “--phred33, --rna-strandness RF, --dta-cufflinks, --un-conc-gz,”. The mapping results were processed by the software Samtools v1.4 [51].

### Identification of lncRNAs

Based on the mapping results, the software Stringtie v1.3.3b [52] was utilized to assemble clean reads to transcripts with the main parameters of “--rf, -e”. Next, the parameter of “merge” was used to merge the assembled transcripts, and remove transcripts with uncertain chain direction and the length within 200 nt. After that, the transcripts were mapped to known databases by the software gffcompare [53] to screen the known transcripts. Finally, protein coding potential of unknown transcripts was predicted with current mainstream analysis methods of CPC2 / Pfam / CNCI [54–56]. Transcripts predicted without potential coding potential simultaneously by above three softwares were defined as novel lncRNA, and transcripts predicted having potential coding potential simultaneously by three softwares were defined as novel mRNA. The genomic features of identified lncRNAs, including the length of transcripts, number of exons and length of open reading frame (ORF), were analysed and compared with mRNAs. The ORFs of known lncRNAs and mRNAs were obtained according to the annotation information in the databases, while the ORFs of novel lncRNAs and novel mRNAs were predicted by the software getorf [57].

### Differential expression analysis

The software Stringtie v1.3.3 [52] was used to estimate expression levels of mRNAs and lncRNAs with the method of FPKM (fragments per kilobase per million mapped reads). Differentially expressed lncRNAs (DELs) and genes (DEGs) were identified by edgeR v3.22.5 [58] with the thresholds of Foldchange ≥ 1 and *pvalue* < 0 0.05. The software clusterProfiler v3.8.1 [59] was used to perform GO (Gene Ontology) [60] and KEGG (Kyoto Encyclopedia of Genes and Genomes) [61] enrichment analysis of DEGs with hypergeometric distribution test. GO terms and KEGG pathways with *pvalue* < 0.05 and gene number ≥ 2 were considered significantly enriched.

### Identification of target genes of DELs

Both co-location and co-expression analyses were performed to predict the target genes of DELs according to the *cis*- and *trans*-acting of lncRNAs. The co-location analysis was based on the physical distance between lncRNAs and the genes in the *P. yessoensis* genome, and DELs that physically overlapped or very close (< 100 kb) to the genes were considered as *cis*-acting of lncRNAs [62]. The co-expression analysis was conducted according to the correlation of expression levels between DELs and mRNAs [63], and genes with the correlation coefficient |r| > 0.95 were predicted as *trans*-regulated target genes. After that, the differentially expressed target genes (DETGs) were identified through Venn analysis between DEGs and the unions of *cis*- and *trans*-regulated target genes. GO and KEGG enrichment analysis of DETGs used the methods as mentioned before. Finally, the interactive relationships between DELs and DETGs were represented by the software Cytoscape [64].

### Expression validation by qRT-PCR analysis

Expression levels of lcnRNAs and genes participating in melanization and biomineralization were validated by qRT-PCR following the way mentioned in [65]. In brief, total RNAs were first isolated utilizing an RNAprep pure tissue kit (Tiangen, China). qRT-PCR was performed with a FastStart Essential DNA Green Master kit (Roche) on a Roche Light Cycler 96 System (Roche). The primers were designed with the software Primer Premier 5.0, and the sequencing information of each primer was provided in Supplementary Table S1. The primer specificity was estimated through blasting them to the genome of *P. yessoensis* with the E-value ≤ 1e-10. Further, PCR melting curves were also analysed to check that a single product was amplified by each primer pair. The gene of *β-actin* was chosen as the reference gene [66]. Three technical replications for each reaction and three individuals for each group as biological replications were utilized here. Finally, the relative expression levels of lncRNAs and genes between healthy and *Polydora*-infected *P. yessoensis* were determined by the 2^−ΔΔCT^ method [67]. Independent t-tests were conducted for significance test of expression differences with the software SPSS version 22.0, and the *p*-value lower than 0.05 was thought as statistical significance.

## Results

### Data processing and lncRNAs identification

**Fig. 2:**
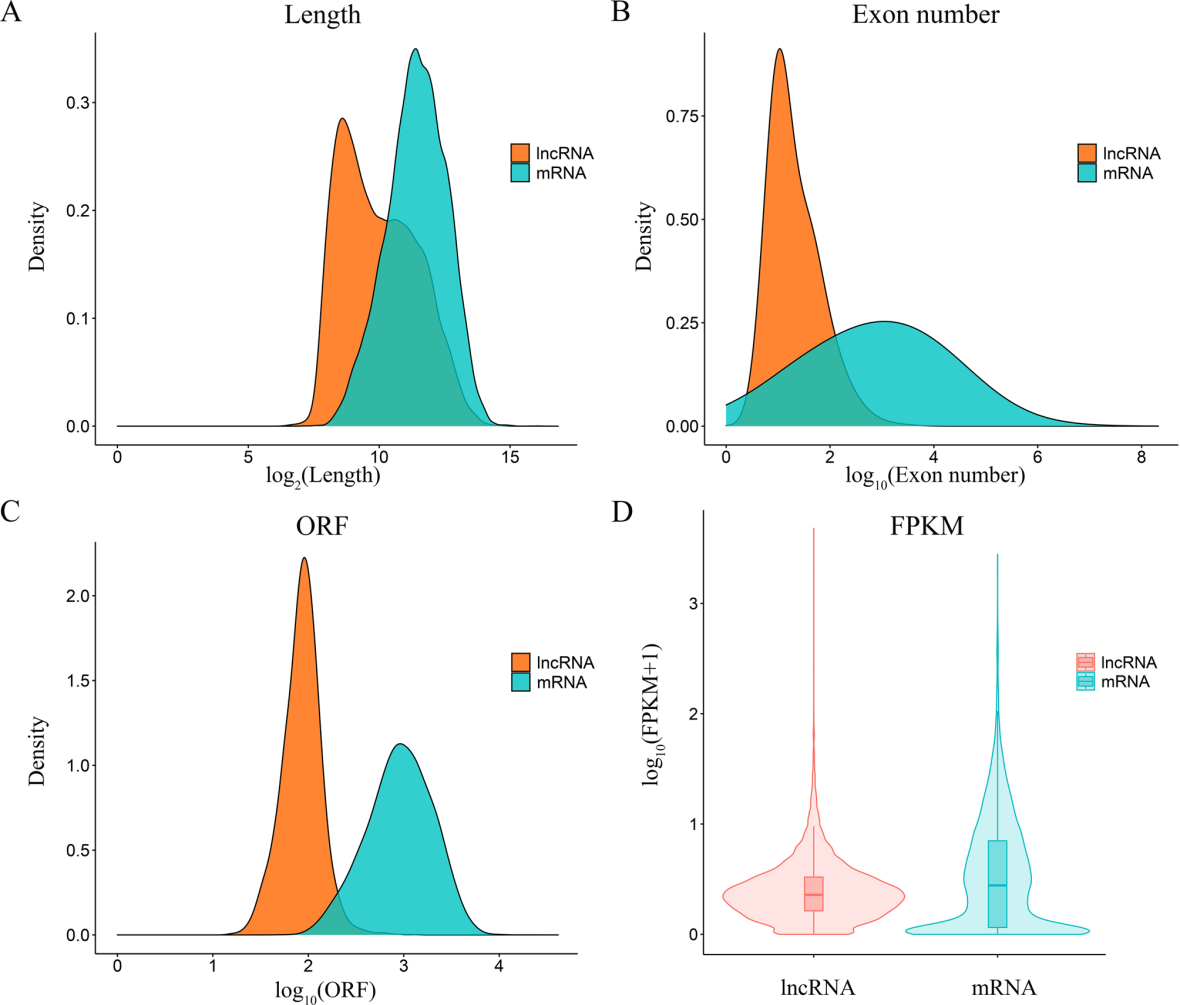
The genomic charaterizations of lncRNAs compared with mRNAs in mantles of *P. yessoensis*. (**A**) Sequence length. (**B**) Number of exons. (**C**) Open reading frame length. (**D**) Expression levels

To identify lncRNAs and mRNAs of *P. yessoensis* involved in defending against *Polydora* infestation, transcriptome sequencing was conducted for the mantles of healthy and diseased individuals. A total of 549,707,542 clean reads with high quality were obtained from the six sequencing libraries with an average of 91,617,924 for each sample. The mapping rate was around 78.07%. The detailed information for the sequencing data was listed in Table S2. In total, 24,659 mRNAs were identified with 24,537 known and 122 new, and 19,133 lncRNAs were identified with 2,203 known and 16,930 new. Among the novel lncRNAs, 48.2%, 35.8%, 11.1% and 5.0% were separately classified as lincRNA (intergenic lncRNAs), sense intronic lncRNAs, sense overlapping lncRNAs and antisense lncRNAs (Figure S1). Compared with mRNAs, lncRNAs generally had shorter sequence length, fewer exon number and shorter ORF lengths, and expressed in a lower level (Fig. 2). PCA (principal component analysis) for the transcript expression level represented individuals from the same group cluster together, and Pearson correlation coefficients within groups was higher than that among groups, which indicated good repeatability of the samples in each group (Figure S2).

### Differentially expressed genes (DEGs) and lncRNAs (DELs) in infested *P. yessoensis*

**Fig. 3:**
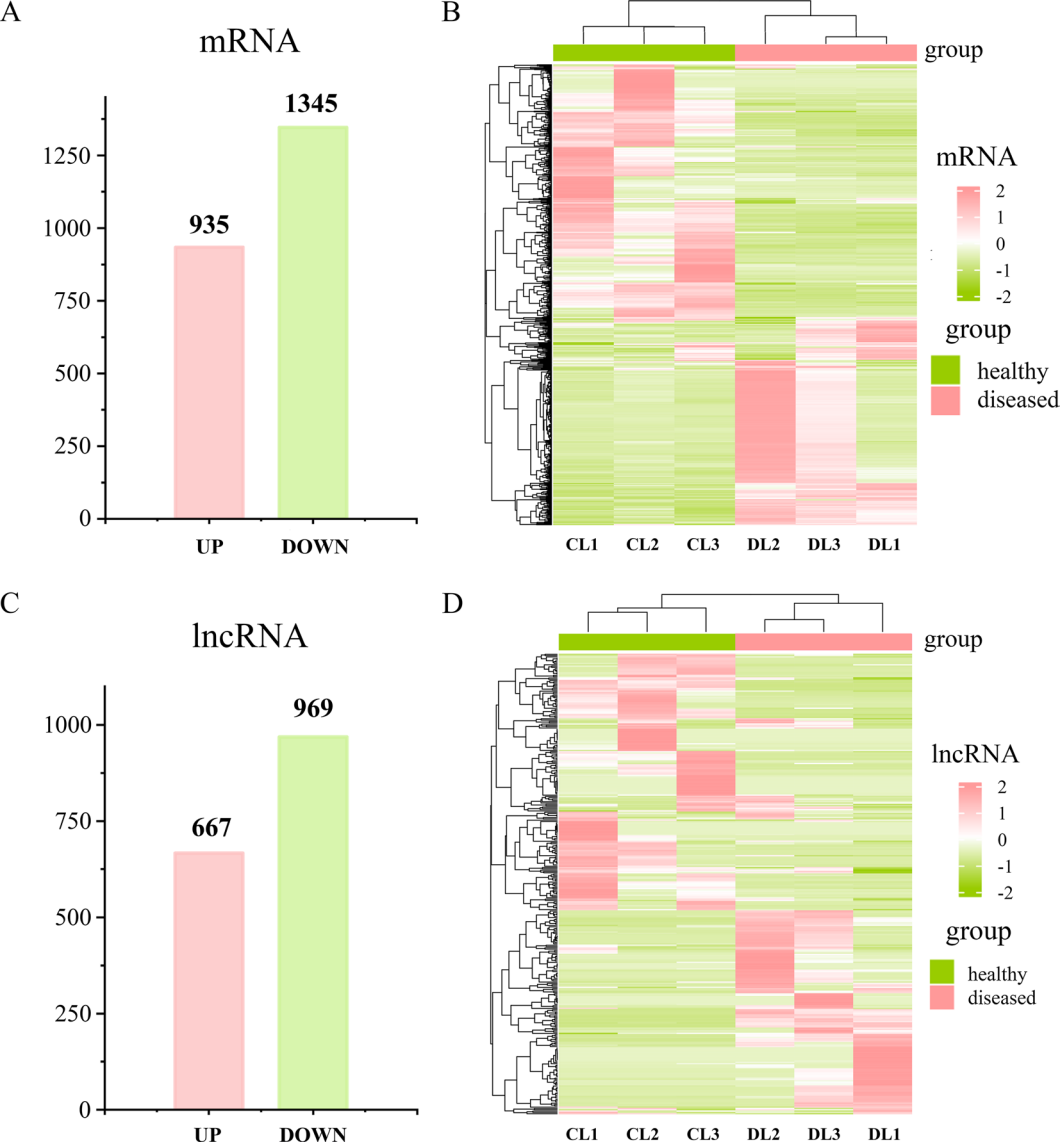
Differentially expressed mRNAs (DEGs) and lncRNAs (DELs) in *Polydora*-infected *P. yessoensis*. Number of up and downregulated DEGs (**A**) and DELs (**C**) in diseased *P. yessoensis* (**B**). Expression profiles of DEGs (**B**) and DELs (**D**) in healthy and diseased *P. yessoensis*

After *Polydora* infestation, there were 2280 genes found regulated in diseased *P. yessoensis*, with 935 upregulated and 1345 downregulated, respectively (Fig. 3A). A total of 1636 differentially expressed lncRNAs were detected in diseased *P. yessoensis*, with 667 upregulated and 969 downregulated, respectively (Fig. 3C). The expression profiles of DEGs and DELs of each sample were displayed in heatmaps, which showed the two groups clustered separately (Fig. 3B and D).

### Functional enrichment analysis for DEGs

**Fig. 4:**
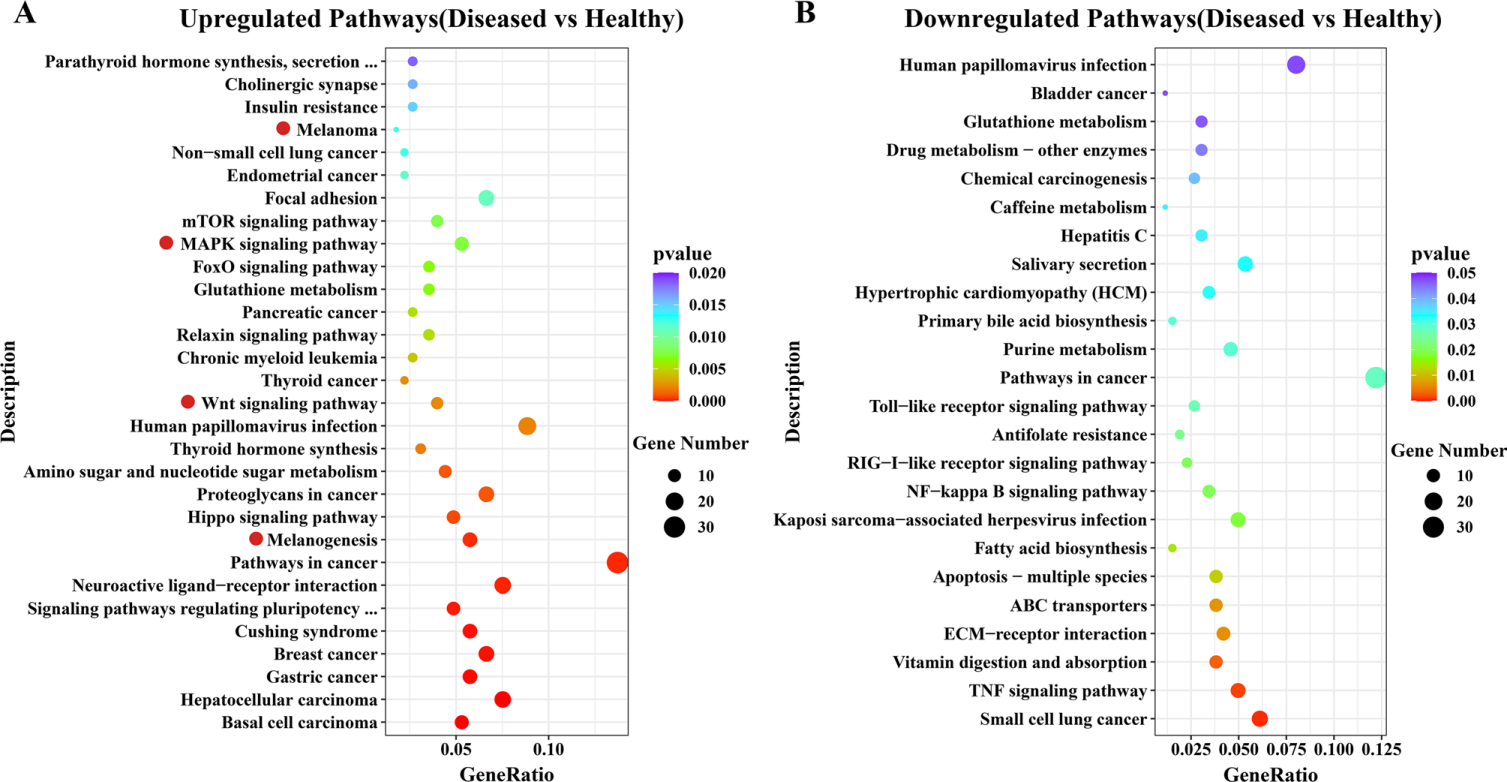
Top 30 Enriched KEGG pathways separately for up (**A**) and downregulated (**B**) DEGs in *Polydora*-infected *P. yessoensis*. Red dots indicate melanization related pathways

GO and KEGG enrichment analyses were separately carried out to explore biological functions of the DEGs. A total of 76 and 47 GO terms were significantly enriched for upregulated and downregulated DEGs, respectively (Table S3). Among those enriched GO functions, terms related to biomineralization and melanization, such as calcium ion binding (GO:0005509), chitin binding (GO:0008061), chitin metabolic process (GO:0006030), copper ion binding (GO:0005507), cell-cell signaling by wnt (GO:0198738) and Wnt signaling pathway (GO:0016055), were discovered significantly upregulated (Table S3). There were 53 and 24 KEGG pathways significantly enriched separately for up and downregulated genes (Fig. 4, Table S4). Obviously, melanization related pathways, i.e., melanogenesis (ko04916), MAPK signaling pathway (ko04010), Wnt signaling pathway (ko04310), cAMP signaling pathway (ko04024) and melanoma (ko05218), were significantly upregulated (Fig. 4A, Table S4). Besides, some immune related pathways were also found enriched, but most in downregulated genes, such as inflammatory mediator regulation of TRP channels (ko04750) and apoptosis (ko04210) enriched in upregulated genes, while pathways of TNF signaling (ko04668), apoptosis-multiple species (ko04215), Toll-like receptor signaling (ko04620), NF-kappa B signaling (ko04064) and RIG-I-like receptor signaling (ko04622) enriched in downregulated genes (Fig. 4B).

### Identification of target genes of DELs and functional enrichment analysis

**Fig. 5:**
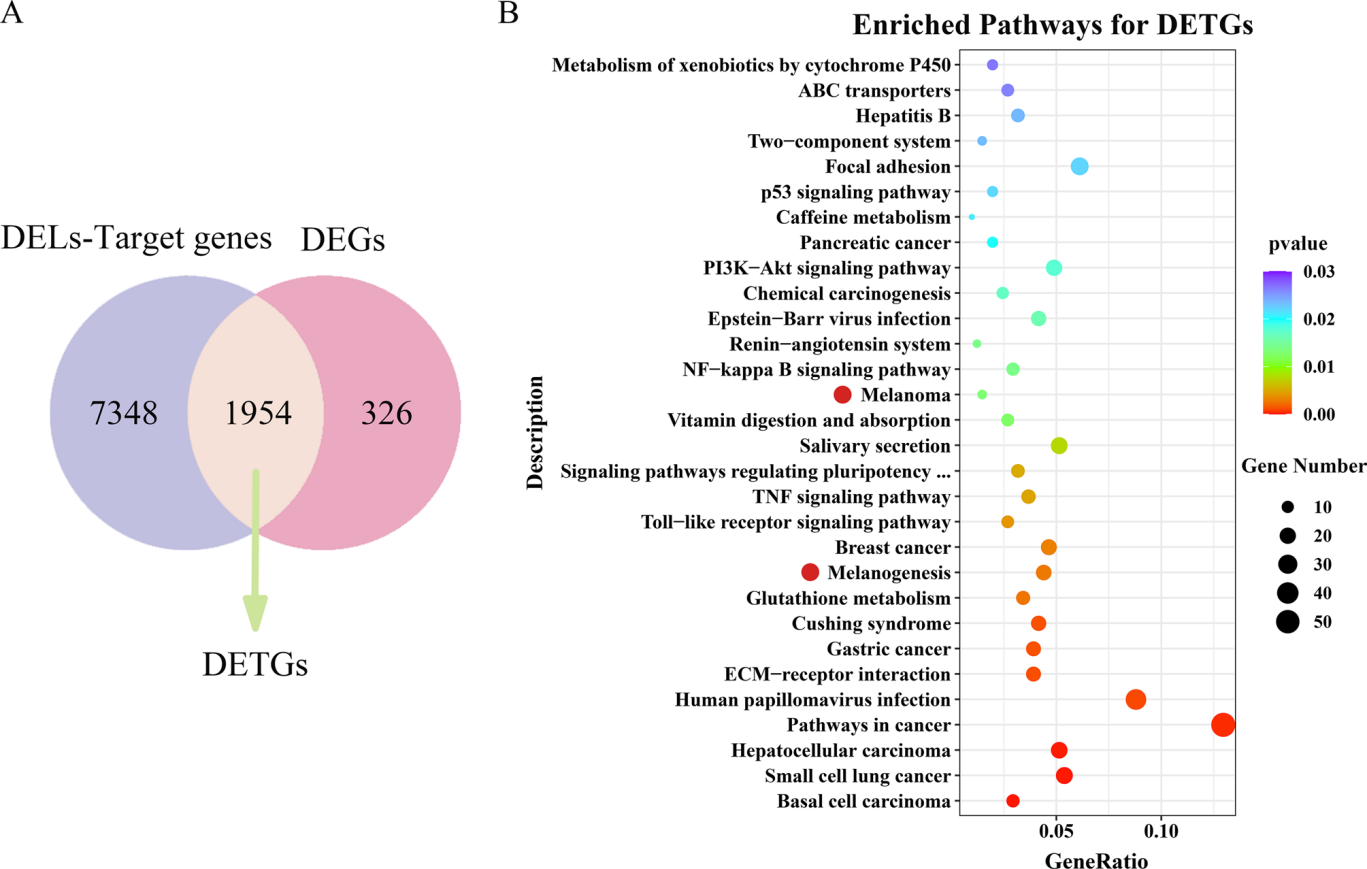
Identification and functional analysis of differentially expressed target genes of DELs (DETGs). (**A**) Venn analysis of target genes of DELs and DEGs. The overlapping indicated the number of DETGs. (**B**) Top 30 Enriched KEGG pathways for DETGs. Red dots indicate melanization related pathways

Co-location and co-expression analyses were separately utilized to identify *cis*- and *trans*-interactions of lncRNAs and mRNAs. After taking the union of two methods, a total of 9302 genes exhibited strong correlations with the 1627 DELs (Fig. 5A). Among these genes, 1954 genes were significantly differentially expressed, which were thought as DETGs (differentially expressed target genes of DELs) (Fig. 5A). GO enrichment analysis for DETGs found 73 GO terms significantly enriched (Table S5), including those associated with biomineralization and melanization, i.e., calcium ion binding (GO:0005509), chitin binding (GO:0008061), chitin metabolic process (GO:0006030), cell-cell signaling by wnt (GO:0198738) and Wnt signaling pathway (GO:0016055). KEGG enrichment analysis for DETGs revealed 44 pathways significantly enriched (Fig. 5B, Table S6). Melanization related pathways, i.e., melanogenesis (ko04916) and melanoma (ko05218), and immune related pathways, e.g., TNF signaling pathway (ko04668), Toll-like receptor signaling pathway (ko04620), NF-kappa B signaling pathway (ko04064) and so on, were also detected significantly enriched. The results revealed the processes of biomineralization and melanization in *P. yessoensis* were regulated by lncRNAs.

### Regulated genes and pathways in melanization and biomineralization

**Fig. 6:**
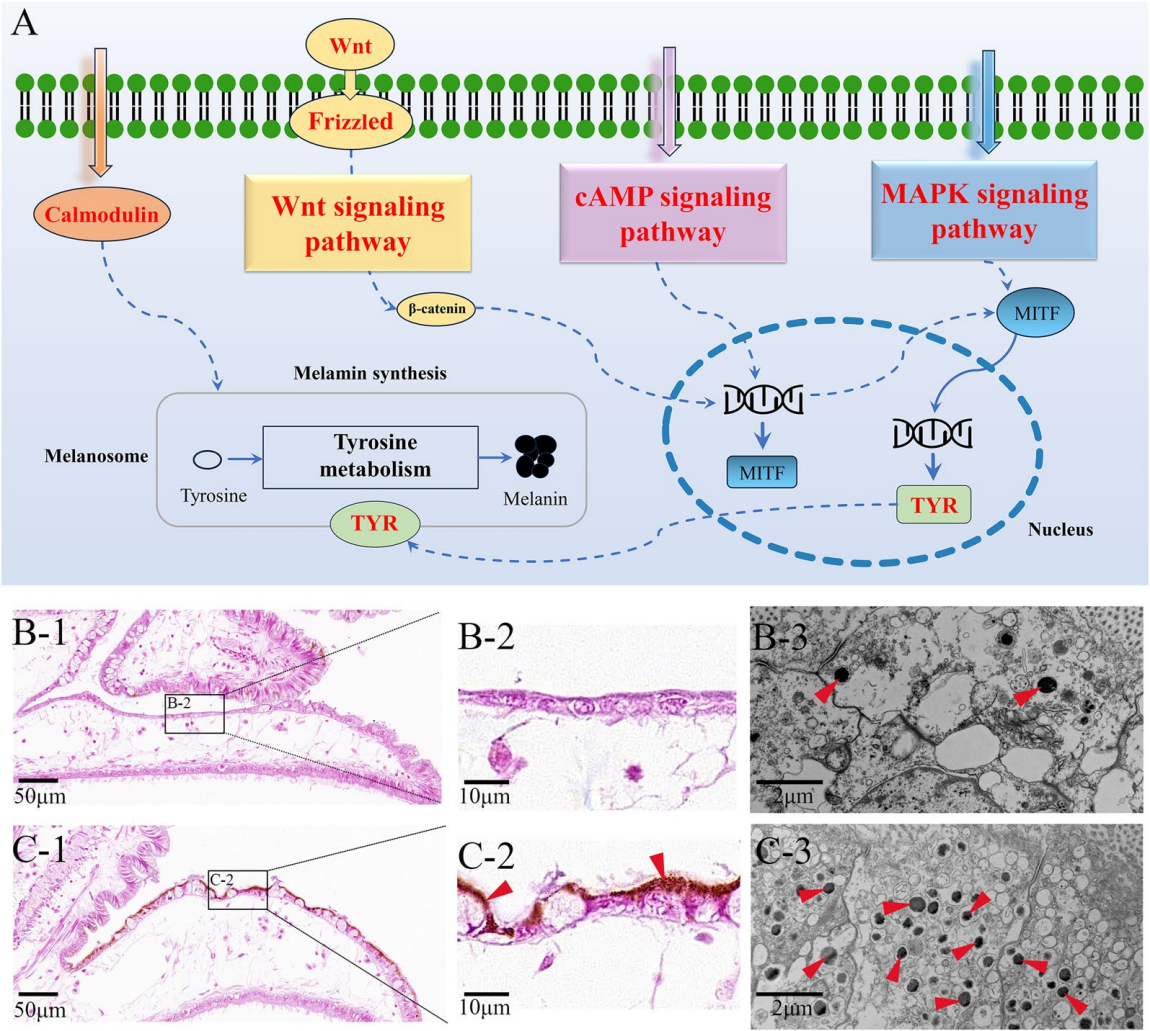
Melanin synthesis in *P. yessoensis*. (**A**) The molecular pathway of melanin synthesis in *P. yessoensis*. (**B**) Melanin granules observed in the mantles of healthy *P. yessoensis* by histological (B-1, B-2) and transmission electron microscope (TEM) techniques (B-3). (**C**) Melanin granules observed in the mantles of *Polydora*-infected *P. yessoensis* by histological (C-1, C-2) and TEM techniques (C-3). Red arrows indicate melanin granules

The pathway of melanogenesis, which exhibits a key role in melanin biosynthesis, was significantly enriched in both upregulated DEGs and DETGs. Regulated genes involved in this pathway were indicated in Fig. 6A, which included *Tyrosinases* (*Tyrs*), *Frizzled*, *Wnts* and *calmodulins*. Three important regulatory pathways for melanin synthesis, i.e., pathways of MAPK signaling (ko04010), Wnt signaling (ko04310) and cAMP signaling (ko04024), were also detected upregulated in DEGs. The results molecularly indicated the increase of melanin formation in diseased *P. yessoensis*. Further, increased melanin granules were also discovered in the middle mantle cells of the diseased individuals under optical microscope and TEM (Fig. 6B C). *Tyr* is the key gene participating in melanin synthesis. There were eight *Tyr* genes (named *Tyr 1–8*) found significantly upregulated in diseased *P. yessoensis*, and they were also involved in DETGs. DETGs related to melanin formation were summarized in Table 1. Besides, many important biomineralization related DETGs were also detected, such as *Pif*s, *perlucin*, *laccase*, *shell matrix protein*, *mucin* and *chitin*s (Table 1).

**Table 1:** Key genes and their regulatory lncRNAs involved in melanization and biomineralization

Gene_ID	Gene description	LncRNA_ ID
**Melanization**		
110,463,428	*Tyr 1*	TCONS_00004873, TCONS_00053074, TCONS_00125024, TCONS_00062895, TCONS_00088543, TCONS_00009210, XR_002463657.1, TCONS_00062902 TCONS_00028231, TCONS_00077335, TCONS_00062898, TCONS_00102934, TCONS_00034553
110,463,665	*Tyr 2*	TCONS_00004873, TCONS_00125024, TCONS_00062895, TCONS_00062898, TCONS_00009155
110,463,673	*Tyr 3*	TCONS_00081590, TCONS_00068583, TCONS_00045834, TCONS_00126106, TCONS_00040660, TCONS_00130012, TCONS_00119057, XR_002462369.1 TCONS_00128950, XR_002464096.1, TCONS_00048096, TCONS_00117145, XR_002463842.1, TCONS_00024464, TCONS_00087657, TCONS_00009155 TCONS_00109522, TCONS_00061795
110,463,689	*Tyr 4*	TCONS_00081590, TCONS_00046855, TCONS_00084794, TCONS_00023420, TCONS_00081589, TCONS_00016124, XR_002464096.1, TCONS_00051920 TCONS_00113324, TCONS_00041417, TCONS_00011307, TCONS_00111152, XR_002462422.1, TCONS_00087657, XR_002463179.1, TCONS_00061430 TCONS_00037395, TCONS_00009155, TCONS_00061795
110,463,699	*Tyr 5*	TCONS_00122484, TCONS_00062897, TCONS_00053074, TCONS_00083009, TCONS_00025471, TCONS_00125024, TCONS_00062895, TCONS_00088543 TCONS_00004873, XR_002463092.1, XR_002463657.1, TCONS_00028231, TCONS_00067511, TCONS_00077335, TCONS_00062898, TCONS_00062903 TCONS_00102934, TCONS_00128849, TCONS_00062904, TCONS_00009155, TCONS_00034553, TCONS_00127775, TCONS_00062902
110,452,398	*Tyr 6*	TCONS_00122484, TCONS_00062897, TCONS_00053074, TCONS_00083009, TCONS_00025471, TCONS_00086968, TCONS_00062900, TCONS_00077335 TCONS_00116040, TCONS_00062902, XR_002463092.1, XR_002463657.1, TCONS_00028231, TCONS_00007665, TCONS_00067511, TCONS_00028230 TCONS_00062903, TCONS_00102934, TCONS_00081614, TCONS_00128849, TCONS_00062904, TCONS_00025258, TCONS_00034553, TCONS_00127775
110,467,506	*Tyr 7*	TCONS_00116040, TCONS_00049064, XR_002461741.1, TCONS_00083009, TCONS_00062896, TCONS_00062900, TCONS_00113324, TCONS_00028230 TCONS_00122484, XR_002463092.1, XR_002463657.1, XR_002462422.1, TCONS_00007665, TCONS_00077335, TCONS_00062903, TCONS_00081614 TCONS_00128849, TCONS_00062901, TCONS_00025258, TCONS_00086831, TCONS_00034553, TCONS_00127775, XR_002461800.1
110,449,337	*Tyr 8*	TCONS_00081590, TCONS_00013147, TCONS_00020916, TCONS_00092959, TCONS_00111152, XR_002463324.1
110,441,941	*Wnt 5b*	XR_002463324.1
110,462,039	*Wnt 1*	XR_002463324.1
110,456,287	*Wn 2b-A*	XR_002463324.1
110,442,897	*Wnt 10a*	TCONS_00005674, TCONS_00028956
110,462,024	*Wnt 6*	TCONS_00122484, TCONS_00062902, TCONS_00062897, TCONS_00053074, TCONS_00083009, TCONS_00025471, TCONS_00125024, TCONS_00062895 TCONS_00004873, TCONS_00088543, TCONS_00098730, XR_002463657.1, TCONS_00028231, TCONS_00067511, TCONS_00077335, TCONS_00062898 TCONS_00102934, TCONS_00128849, TCONS_00062904, TCONS_00034553
110,443,365	*Wnt 16*	TCONS_00116040, TCONS_00062896, TCONS_00016124, TCONS_00113324, XR_002462422.1, TCONS_00007665, XR_002463179.1, TCONS_00058323 TCONS_00028230, TCONS_00081614, TCONS_00062901, TCONS_00127775
110,447,918	*calmodulin*	TCONS_00111362, TCONS_00111360, TCONS_00057975, TCONS_00112054, TCONS_00005673, TCONS_00005680, XR_002464796.1
110,443,533	*calmodulin*	TCONS_00126771
110,465,124	*calmodulin*	TCONS_00094494
110,464,711	*Frizzled 9*	TCONS_00078335, TCONS_00084794, TCONS_00092959, TCONS_00002551, TCONS_00081589, TCONS_00113324, XR_002462422.1
**Biomineralization**		
110,453,492	*Pif 1*	XR_002461890.1, TCONS_00116833
110,440,284	*Pif 7*	TCONS_00081590, XR_002463842.1, XR_002461905.1, TCONS_00011307, TCONS_00024464, TCONS_00087657, XR_002463179.1, TCONS_00061430 TCONS_00128950, TCONS_00109522, TCONS_00061795, TCONS_00051920, TCONS_00048096, XR_002462369.1, XR_002464096.1, TCONS_00016124 TCONS_00068583, TCONS_00046855, TCONS_00045834, TCONS_00040660, TCONS_00130012, TCONS_00023420, TCONS_00117145
110,457,669	*shell matrix protein*	TCONS_00081590, TCONS_00046855, TCONS_00023420, TCONS_00081589, TCONS_00016124, XR_002462369.1, XR_002464096.1, TCONS_00051920 TCONS_00128950, TCONS_00011307, TCONS_00111152, XR_002462422.1, TCONS_00087657, XR_002463179.1, TCONS_00061430, TCONS_00037395 TCONS_00061795, TCONS_00041417
110,445,403	*Chitin*	TCONS_00128950, TCONS_00068583, TCONS_00045834, TCONS_00126106, TCONS_00040660, TCONS_00130012, XR_002462369.1, XR_002464096.1 TCONS_00048096, TCONS_00117145, XR_002463842.1, TCONS_00024464, TCONS_00087657, TCONS_00031534, XR_002463765.1, TCONS_00119057 TCONS_00038196, TCONS_00109522, TCONS_00061795
110,457,124	*Chitin*	TCONS_00116040, TCONS_00046855, TCONS_00084794, TCONS_00081589, TCONS_00016124, TCONS_00113324, TCONS_00011307, XR_002462422.1 XR_002463179.1, TCONS_00061430, TCONS_00028230, TCONS_00081614, TCONS_00127775
110,457,103	*Chitin*	TCONS_00116040, TCONS_00083009, TCONS_00062896, TCONS_00093573, TCONS_00062900, TCONS_00077335, TCONS_00061374, XR_002463092.1 TCONS_00122484, XR_002463657.1, TCONS_00061373, XR_002462422.1, TCONS_00007665, TCONS_00058323, TCONS_00028230, TCONS_00062903 TCONS_00081614, TCONS_00128849, TCONS_00062901, TCONS_00086831, TCONS_00034553, TCONS_00127775
110,457,672	*Chitin*	TCONS_00116040, TCONS_00062902, TCONS_00062897, TCONS_00083009, TCONS_00025471, TCONS_00062900, TCONS_00062899, TCONS_00077335 TCONS_00122484, XR_002463092.1, XR_002463657.1, TCONS_00028231, TCONS_00007665, TCONS_00067511, TCONS_00028230, TCONS_00062903 TCONS_00102934, TCONS_00081614, TCONS_00128849, TCONS_00062904, TCONS_00034553, TCONS_00127775
110,442,859	*Laccase*	TCONS_00081590, TCONS_00046855, TCONS_00081589, TCONS_00016124, TCONS_00115897, TCONS_00113324, TCONS_00041417, TCONS_00011307 XR_002462422.1, XR_002463179.1, TCONS_00061430, TCONS_00037395, TCONS_00061795
110,442,757	*Perlucin*	TCONS_00116040, TCONS_00046855, TCONS_00062896, TCONS_00092959, TCONS_00002551, TCONS_00081589, TCONS_00016124, TCONS_00115897 TCONS_00048752, TCONS_00113324, XR_002462422.1, TCONS_00007665, XR_002463179.1, TCONS_00061430, TCONS_00090766, TCONS_00037395 TCONS_00058323, TCONS_00000279, TCONS_00028230, TCONS_00091397, TCONS_00081614, TCONS_00062901, TCONS_00127775
110,451,253	*Mucin*	TCONS_00116040, TCONS_00083009, TCONS_00084794, TCONS_00113324, XR_002463092.1, XR_002462422.1, TCONS_00038234, TCONS_00028230 TCONS_00081614, TCONS_00038231, TCONS_00124179, TCONS_00127775, XR_002461800.1

### Core lncRNA-mRNA networks construction

**Fig. 7:**
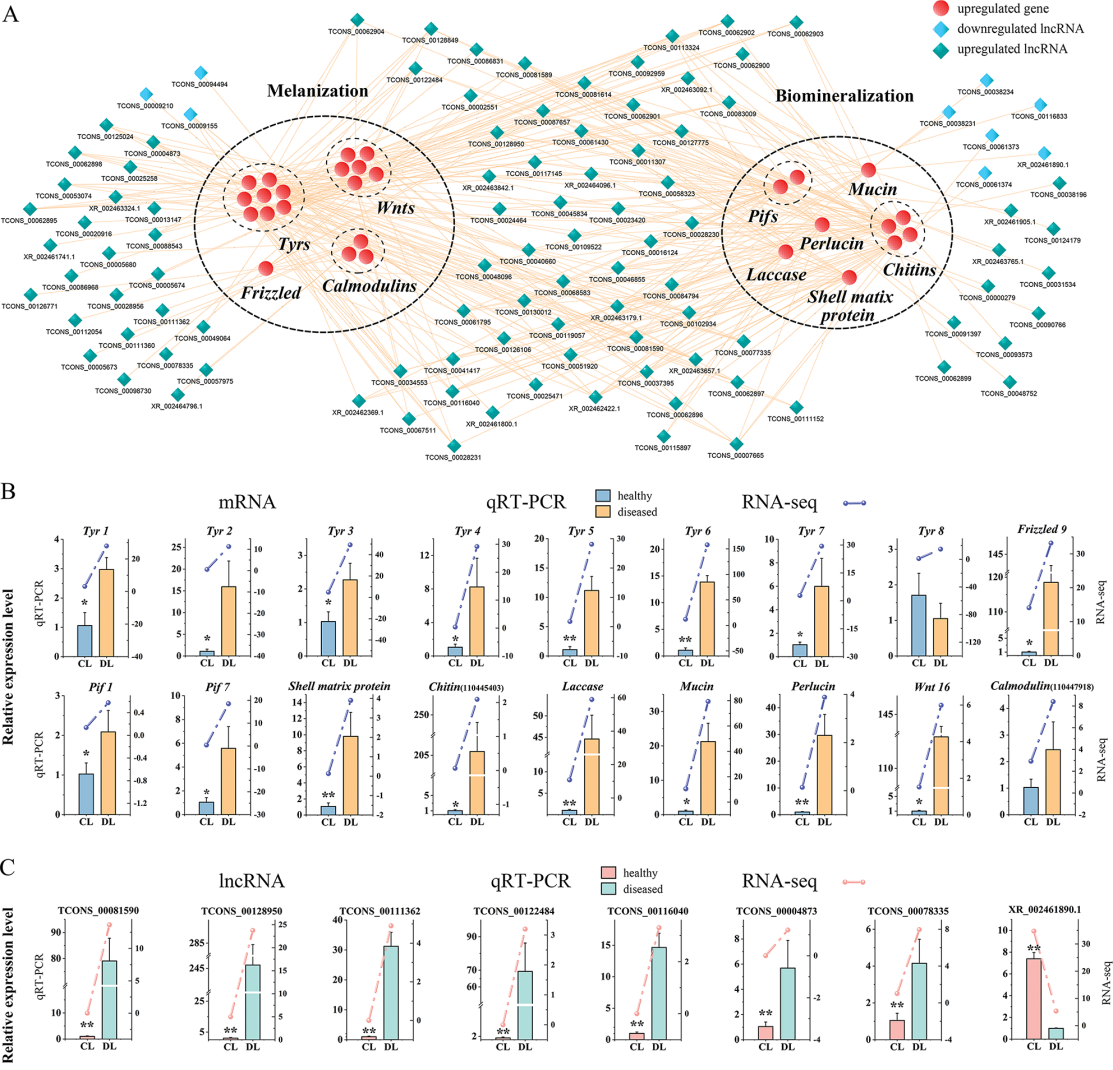
The lncRNA-mRNA interactive network involved in melanization and biomineralization. (**A**) The core lncRNA-mRNA network. (**B**) Expression validation of mRNAs involved in the network by qRT-PCR. (**C**) Expression validation of lncRNAs involved in the network by qRT-PCR. Vertical bars represent mean ± standard error (N = 3); * stands for *p-*value ≤ 0.05, ** stands for *p-*value ≤ 0.01

Integrated analysis of the lncRNA-mRNA interactions involved in melanization and biomineralization were performed to construct a core lncRNA-mRNA network. As a result, a total 352 interactive lncRNA-mRNA pairs including 105 DELs and 28 DETGs were identified (Fig. 7A). Some lncRNAs were simultaneously corresponding to multiple mRNAs in the network. For example, TCONS_00116040 were predicted to regulate *wnt 16*, *tyr6*, *tyr7*, *chitin* (110,457,672), *perlucin* and *mucin*. Besides, most genes showed to be regulated by multiple lncRNAs. For example, *Tyr 1* was the potential target gene of 13 different lncRNAs (Table 1). Expression levels of eighteen key genes in the network, including eleven involved in melanin formation and seven involved in shell formation, were further verified by qRT-PCR, expression patterns of which displayed highly consistent with RNA-seq (Fig. 7B). Eight lncRNAs were randomly selected from the network for validation, and the qRT-PCR expression patterns also showed high consistency with the RNA-seq data (Fig. 7C).

## Discussion

In the present study, lncRNAs and mRNAs triggered in mantles of *Polydora*-infected *P. yessoensis* were first examined and analyzed to explore the molecular regulatory mechanisms of *P. yessoensis* responding to *Polydora* infestation. The genomic characterizations of lncRNAs in *P. yessoensis* mantles is similar to that observed in other species [39, 42, 43, 68], which exhibited shorter sequence and ORF length, fewer number of exons and lower expression levels than mRNAs. Unlike mRNAs, the sequence conservation of lncRNAs is generally low [24, 68]. Here, over 88% of *P. yessoensis* lncRNAs were novel identified, which had no orthologs in other species. High ratio of novel lncRNAs were also found in other marine organism, for example, 98% lncRNAs identified in the spleen of the flounder (*Paralichthys olivaceus*) were unknown [68], and lncRNAs obtained in the gonad of the Pacific oyster were all novel [40]. Though the lncRNA resources in molluscs are still limited, the high ratio of unknown lncRNAs found in this study approved the view that majority of lncRNAs were short of primary sequence conservation [24, 68].

In invertebrates, melanizaiton of injured tissues and pathogens is an important innate defense process [69]. In molluscs, it is common in the process of shell coloration. Moreover, evidence also indicated significant roles of melanizaiton in encapsulation of non-self-entities and wound healing in molluscs [13]. The mantle tissue, an evolutionarily homologous organ and responsible for the shell formation, was shown to mainly support melanization, especially the external epithelium of the mantle [11, 13]. After *Polydora* infestation, melanistic inner shell layers was a major phenotypic trait in diseased individuals (Fig. 1). Moreover, obviously increased melanin granules were observed in epithelial cells of the edge mantle in diseased scallops by histological and TEM study. Further, genes involved in melanin synthesis were significantly upregulated in *Polydora*-infected *P. yessoensis*. Thus, the current work demonstrated the crucial role of melanizaiton for *P. yessoensis* in resisting against *Polydora* infestation simultaneously at the phenotypic, cellular, and molecular levels.

In this study, there were a total of 2280 genes and 1636 lncRNAs found differentially expressed between healthy and *Polydora*-infected *P. yessoensis*. Functional enrichment analysis revealed that melanin biosynthesis related pathways, i.e., melanogenesis (ko04916), MAPK signaling pathway (ko04010), Wnt signaling pathway (ko04310), cAMP signaling pathway (ko04024) and melanoma (ko05218), were obviously enriched among upregulated DEGs. Melanogenesis is a key molecular pathway controlling the synthesis of melanin, containing complex multistep reactions to transform tyrosine to final melanin [70, 71]. Wnt, MAPK and cAMP signaling pathways are three main regulatory pathways for melanin synthesis [71]. The obvious upregulation of these pathways indicated the acceleration of melanin production in diseased *P. yessoensis*. To further elucidate the molecular regulatory mechanism of this process, lncRNAs involved in the synthesis of melanin were screened by association analysis. Notably, the pathway of melanogenesis (ko04916) were also obviously enriched in DETGs, implying a very close correlation between lncRNAs and melanin biosynthesis.

Tyrosinase (Tyr), one kind of phenoloxidases, undergoes evident gene expansion in the genome of molluscs [72], which exerts great influences on diverse molluscan biological processes, including melanogenesis, biomineralization, and immune response [73–79]. It is a crucial and rate-determining enzyme in the process of melanogenesis, catalyzing tyrosine oxidated to dopaquinone [71, 80]. Emerging evidence in molluscs suggests the expression of *Tyrs* is regulated by lncRNAs. In the Pacific oyster, two members of *Tyrs* were differentially expressed between different shell color individuals, which were associated with melanin biosynthesis, and they were the target genes of nine *cis-acting* lncRNAs [42]. Similarly, four *Tyrs* with their eleven corresponding *cis*-acting lncRNAs were detected modulating melanin biosynthesis in *R. philippinarum* [45]. In this study, a total of eight *Tyrs* showed significantly higher expression levels in diseased *P. yessoensis*, indicating their important roles in *P. yessoensis* to defend against *Polydora* infestation. By interactive analysis, 72 different DELs (such as TCONS_00004873, TCONS_00081590, TCONS_00128950, TCONS_00122484, TCONS_00116040, etc.) were predicted to regulate the expressions of the eight *Tyr* genes (Table 1), which could be key candidate lncRNAs participating in the regulatory process of melanin synthesis in *P. yessoensis*. Wnt signaling pathway is one of main upstream regulatory pathways for melanin synthesis [71] In canonical Wnt signaling pathway, the binding of Frizzled and Wnt results in the suppression of GSK 3*β* (glycogen synthase kinase 3*β*) and the accumulation of *β*-catenin [81]. Subsequently, *β*-catenin will be transported to the nucleus and activates the transcription of MITF (microphthalmia-associated transcription factor), which regulates the expression of *Tyr* and finally regulates the melanin synthesis [81]. LncRNAs participating in the regulation of Wnt signaling pathway that affects melanin synthesis has been reported in vertebrates (such as human, mouse, goat, fish and so on) [25, 82–84], but has seldom been reported in molluscs. In the present study, some members of the Wnt signaling pathway, i.e., *Frizzled 9* and *Wnts*, were found upregulated by their corresponding DELs in diseased scallops (Table 1), which were probably involved in the modulation of melanin synthesis. Therefore, interactions between the above core genes and their corresponding DELs possibly promoted the biosynthesis of melanin, which would be then transported to shells and encapsulate the *Polydora*.

Sometimes, melanization of foreign entities goes with a biomineralization process to embed melanistic intruders in a newly formed shell layer, just as observed in diseases of ROD and BRD [13–15]. In the present study, a variety of mineralized protuberances formed on the shell inner surface to prevent further invasion of *Polydora*. This probably resembles processes of pearl formation and nacrezation, which is actually a common response to irritants (such as abiotic entities, pathogens and parasites) in molluscs [13]. Some biomineralization-related genes, such as *Pif*s, *perlucin*, *laccase*, *shell matrix protein*, *mucin* and *chitin*s, were found significantly upregulated in *Polydora*-infected individuals, probably indicating the accelerated secretion and rearrangement of shell matrix to repair the damaged shell [13–16]. Pif, an acid shell matrix protein, has been demonstrated as a crucial macromolecule involved in the nacreous layer formation in molluscs [85]. Pif is deemed to participate in the initiation of aragonite crystallization and subsequently stack aragonite tablets in the nacreous layer [85–88]. In our previous study, 8 *Pif* homologs in total were identified in the genome of *P. yessoensis*, and most of these genes represented dramatically increased expression levels in the edge mantle tissues of scallops infected by *Polydora*, indicating their important function in shell formation [65]. In this study, two of the *Pifs* (*Pif1* and *Pif7*) were detected significantly upregulated in the mantle transcriptome of diseased individuals, consistent with the expression trends in our previous study. The expression of *Pif1* was predicted to be regulated by two DELs of XR_002461890.1 and TCONS_00116833, and *Pif7* was the potential target gene of 23 DELs (such as TCONS_00081590, TCONS_00128950, TCONS_00046855, etc.). Therefore, these lncRNAs probably took part in the nacreous layer formation by regulating the expression of *Pifs* to repair the damaged shells and resist the further *Polydora* invading.

Perlucin, a C-type lection domain protein, was reported to promote the nucleation and growth of calcium carbonate crystals during shell formation in molluscs [89–91]. Besides, it was also thought to perform important functions as an organic support in biomineralization [13, 91]. Several perlucin-like transcripts were found upregulated in the mantles and extrapallial fluids of BRD clams [16]. Herein, a *perlucin* also displayed an obviously higher expression level in *Polydora*-infected *P. yessoensis*, and it was potentially targeted by 23 DELs (e.g., TCONS_00116040, TCONS_00046855, TCONS_00062896, etc.). Laccase, another phenoloxidases, has been shown participating in shell immunity, pigmentation and biomineralization [8, 92, 93]. During biomineralization, laccase could direct the cross-linking of shell matrix [93, 94]. A significantly upregulated expression level of a *laccase* gene was discovered in BRD clams [16]. Similar with the result in clams, a *laccase* in *Polydora*-infected *P. yessoensis* also represented an obviously higher expression level than that in healthy individuals, and the *laccase* gene was targeted by 13 DELs (e.g., TCONS_00081590, TCONS_00046855, XR_002463179.1, etc.), suggesting their important role in biomineralization process. There were also some other biomineralization directly related genes, such as *shell matrix protein-like*, *mucin* and *chitin*, with their corresponding lncRNAs detected to be regulated. The above biomineralization associated genes were probably core genes controlling shell formation in *P. yessoensis*, and their expressions could be regulated by lncRNAs, which would have great influence on *P. yessoensis* to respond to *Polydora* infestation.

## Conclusion

In the present study, a high-throughput transcriptome analysis was conducted in the mantles of the healthy and *Polydora*-infected *P. yessoensis* by RNA sequencing. The study provided the first genome-wide lncRNAs catalog in the mantles of *P. yessoensis*. The primary genomic features of lncRNAs were systematically investigated. Compared with mRNAs, lncRNAs showed shorter sequence and ORF lengths, fewer number of exons and lower expression levels. In total, 2280 DEGs and 1636 DELs were detected in *Polydora*-infected individuals. Functional enrichment analysis revealed that DEGs involved in melanization and biomineralization were significantly upregulated, further, obviously increased melanin granules were observed in epithelial cells of the edge mantle in diseased scallops by histological and TEM study, indicating the crucial role of melanizaiton and biomineralization in *P. yessoensis* to resist against *Polydora* infestation. Moreover, many key DEGs, such as *Tyrs*, *Frizzled*, *Wnts*, *calmodulins*, *Pifs*, *perlucin*, *laccase*, *shell matrix protein*, *mucins* and *chitins*, were predicted to be targeted by DELs. Finally, a core lncRNA-mRNA interactive network involved in melanization and biomineralization was constructed and validated by qRT-PCR. This work provides valuable resources for the study of lncRNAs in scallops, and adds a new insight into molecular regulatory mechanisms of *P. yessoensis* responding to *Polydora* infestation, that will contribute to *Polydora* disease control and breeding of disease-resistant varieties in molluscs.

### Electronic supplementary material

Below is the link to the electronic supplementary material.

Supplementary Material 1: Supplementary Figures and Tables S1-S2

Supplementary Material 2: Supplementary Tables S3-S4

Supplementary Material 3: Supplementary Tables S5-S6

## Data Availability

All the sequencing raw data of RNA-seq have been deposited into the NCBI SRA database with the accession number of PRJNA1025318. The datasets generated during this study are included in the article and its supplementary information files.

## Abbreviations

ncRNA: non-coding RNA
rRNA: ribosomal RNA
lncRNA: long non-coding RNA
lincRNA: intergenic lncRNA
DEG: differentially expressed gene
DELs: differentially expressed lncRNAs
DETG: differentially expressed target gene
ORF: open reading frame
PCA: principal component analysis
TEM: transmission electron microscope
*Tyr*: *Tyrosinase*
qRT-PCR: quantitative real-time PCR
GO: Gene Ontology
KEGG: Kyoto Encyclopedia of Genes and Genomes
