# Supplementary material for: Transcriptome analysis reveals core lncRNA-mRNA networks regulating melanization and biomineralization in Patinopecten yessoensis shell-infested by Polydora
Source: BMC Genomics. 2023 Nov 29;24:723. doi: 10.1186/s12864-023-09837-w (PMC10687851; doi:10.1186/s12864-023-09837-w)
Supplement: Supplementary file 1 — Supplementary Material 1: Supplementary Figures and Tables S1-S2 [file 12864_2023_9837_MOESM1_ESM.docx]

**Supplementary Figures:**


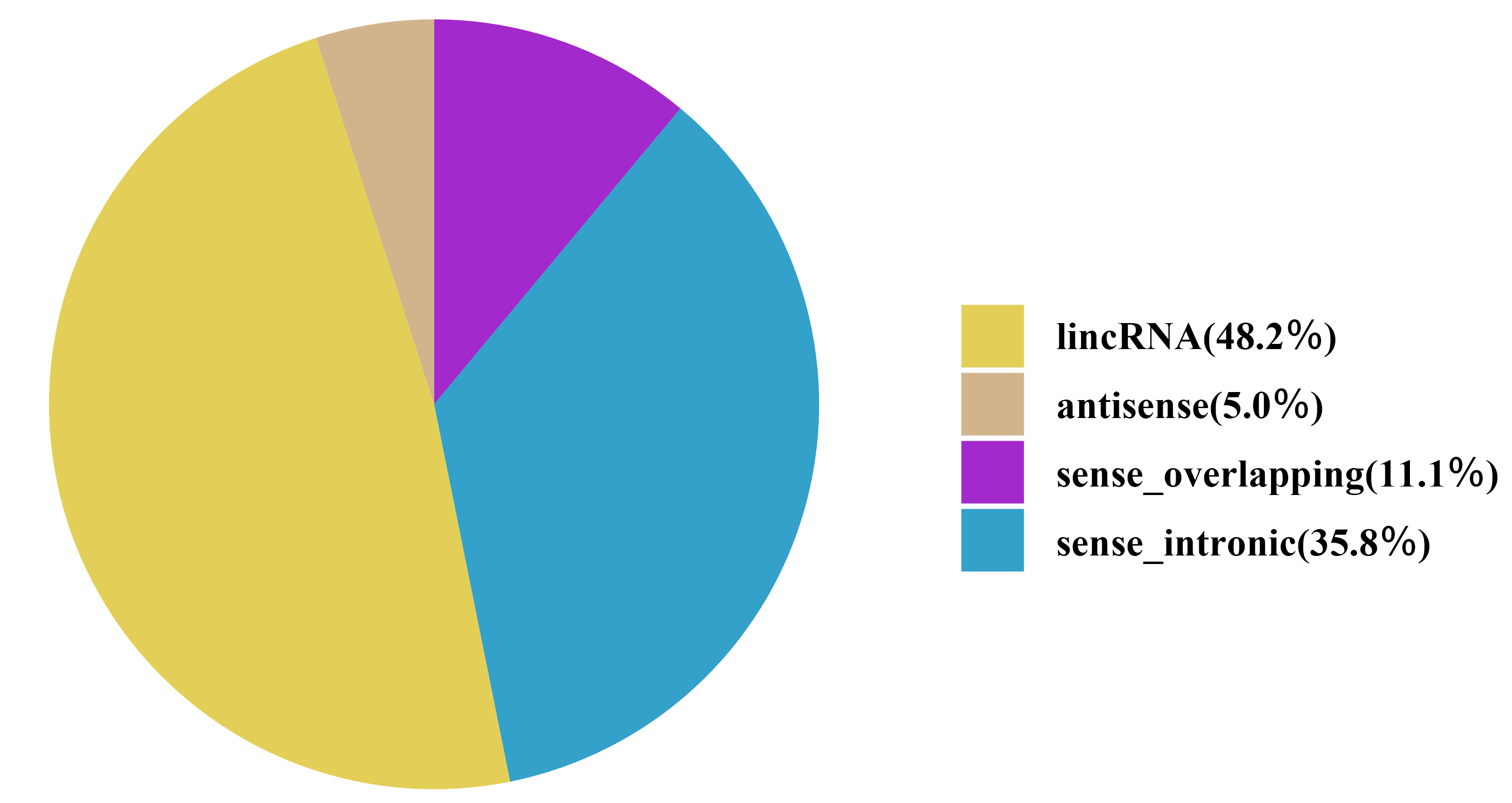


Supplementary Figure S1. The classification of lncRNAs identified in the mantles of *P. yessoensis*.


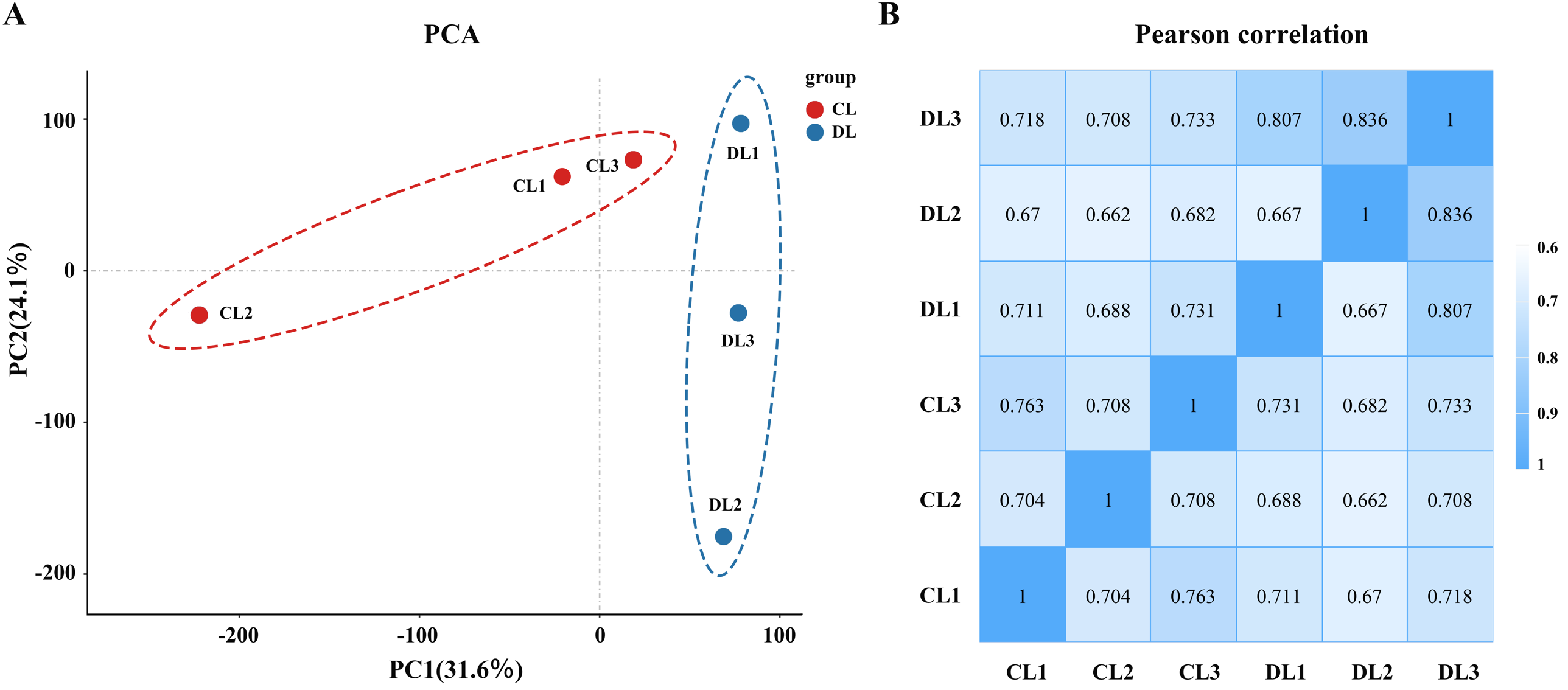
 Supplementary Figure S2. Transcript expression correlations among different samples of healthy and *Polydora*-infected *P. yessoensis*. A. Principal component analysis of the transcript expression levels. B. Pearson correlation coefficients among different samples.

**Supplementary Tables:**

Table S1. Information of primers used in the present study.

| **Primer name** | **Gene_ID** | **Description** | **Primer sequences (5’-3’)** |
| --- | --- | --- | --- |
| TYR-1-F | 110463428 | *Tyr 1* | GCTGATCCCGTGTTCTATTGTCTC |
| TYR-1-R |  |  | TCCATATTGTCTCTGCTTGTGTCTG |
| TYR-2-F | 110463665 | *Tyr 2* | CGATGGCTAGAATGGCTGGAATG |
| TYR-2-R |  |  | GCTACTGTTTGCTGGGCTCTTG |
| TYR-3-F | 110463673 | *Tyr 3* | CATCTACGCTCCACCGCCTAG |
| TYR-3-R |  |  | GCTCCTCTTCTTGATACGCATCTTC |
| TYR-4-F | 110463689 | *Tyr 4* | GCACCGTCGCCAACTTGTC |
| TYR-4-R |  |  | CCATCGCCATACCACCTCCTC |
| Tyr-5-F | 110463699 | *Tyr 5* | CGGTGACGGAGTGGTTAATACAG |
| Tyr-5-R |  |  | GGTTGATGATGTTGTTGATGAGACG |
| TYR-6-F | 110452398 | *Tyr 6* | GCCGAACCTACCGACTCTATGC |
| TYR-6-R |  |  | ACATAAGACTGCCACCGCTTCC |
| TYR-7-F | 110467506 | *Tyr 7* | ATGTCGTGTCCAATCAGAAGAGAAC |
| TYR-7-R |  |  | TAGACTGGCACTCCGAGAACAAG |
| TYR-8-F | 110449337 | *Tyr 8* | GCAGAATGGCGTCAGGTCAC |
| TYR-8-R |  |  | GGCAGCATCGTTGTGGAAGTC |
| Wnt-F | 110443365 | *Wnt 16* | TGACTGACTGCTCGTGCGATAC |
| Wnt-R |  |  | TTTGCTAAAGGACACACCGTAATGG |
| CaM-F | 110447918 | *Calmodulin* | CAGAATCACCGCCTCAGAACTTG |
| CaM-R |  |  | CCGTTTCCGTCAGCATCTACTTC |
| Frizzled-F | 110464711 | *Frizzled 9* | TGGTTCTTGGCAGCAGGTAGG |
| Frizzled-R |  |  | GGTCAGTTCGTCTCCGTCCAC |
| PIF-1-F | 110453492 | *PIF 1* | TGGCGACTGTGACAACGACTAC |
| PIF-1-R |  |  | TGCGTTTCGTGCTCGTCCTC |
| PIF-7-F | 110440284 | *PIF 7* | CGAAGCAAGAAGCAACGAGAGG |
| PIF-7-R |  |  | GACGGGCGGCACATAAAGAC |
| Shell matrix-F | 110457669 | *Shell matrix protein* | GGGAGCGTGGAAATCGTCATAC |
| Shell matrix-R |  |  | GGACTGTGGCTGATATGGAATGTG |
| Chitin-F | 110445403 | *Chitin* | CCTCGTTCTCACCATCGCATTG |
| Chitin-R |  |  | GCCTGTGTCGTTGTCGGTATTATG |
| Laccase-F | 110442859 | *Laccase* | GAAGACGATGGGTATGGAATGAAGG |
| Laccase-R |  |  | GCGAAGGTGTAGAATGTAAGCAATG |
| Perlucin-F | 110442757 | *Perlucin* | TGGTGAGATGTGGGACTGGATG |
| Perlucin-R |  |  | GTCTGCTAGGTAGGAGTTCTTGTTC |
| Mucin-F | 110451253 | *Mucin* | CAACAACCACGGAAGACATTACAAC |
| Mucin-R |  |  | CGGCTCATCCATTGGCATAGAC |
| LOC977-OT2-F | TCONS_00081590 |  | TCTTGCAGACATGAGGTGTGTTTGG |
| LOC977-OT2-R |  |  | TTCTTCAGAGATACAACGGCTGGTG |
| LINC8034-F | TCONS_00128950 |  | GGACCAGCAGACAAGACCAAGG |
| LINC8034-R |  |  | TTACTTCCCATGAGACGAGCGAATG |
| LINC5910-F | TCONS_00111362 |  | ATACAGGACATTGGTACGTATGTTG |
| LINC5910-R |  |  | TCTGTCAACAGACATACCAATATCC |
| LIN7632-F | TCONS_00122484 |  | GTGACTGTGTTGTGAGCCAAATGC |
| LIN7632-R |  |  | TGTGGAATCGCCATACATGCTAAGG |
| LINC7045-F | TCONS_00116040 |  | GTAGAGGCGAATGTCCAGCAGAAG |
| LINC7045-R |  |  | TCCAGACTATCCAGGGTGTACTTCC |
| LINC260-F | TCONS_00004873 |  | CTGTTCACCTGTTACTGGGCTATC |
| LINC260-R |  |  | ACTCCAGTGGTAGGTGTACAGATAC |
| LINC4269-F | TCONS_00078335 |  | GTGAAGTGGACAGTAGTGGACAGG |
| LINC4269-R |  |  | GGTTCCTCAAGAGTCTGCGATAGTG |
| XR-461890.1-F | XR_002461890.1 |  | GCAACAGGACAACGGCTCAG |
| XR-461890.1-R |  |  | AAGCACGGAAGAGGAGTACAGG |

Table S2. Summary of the transcriptome sequencing data.

| **Sample** | **RawReads** | **RawBases** | **CleanReads** | **CleanBases** | **Q20** | **Q30** | **Mapping Rate** |
| --- | --- | --- | --- | --- | --- | --- | --- |
| **CL1** | 108,000,000 | 16.18 Gb | 103,000,000 | 15.51 Gb | 97.35 | 92.95 | 78.07% |
| **CL2** | 96,102,252 | 14.42 Gb | 91,560,810 | 13.73 Gb | 97.22 | 92.75 | 73.44% |
| **CL3** | 91,334,314 | 13.70 Gb | 87,923,228 | 13.19 Gb | 97.36 | 92.83 | 78.47% |
| **DL1** | 95,996,418 | 14.40 Gb | 92,059,238 | 13.81 Gb | 97.37 | 92.9 | 79.18% |
| **DL2** | 95,629,968 | 14.34 Gb | 91,863,726 | 13.78 Gb | 97.52 | 93.2 | 77.78% |
| **DL3** | 87,709,366 | 13.16 Gb | 83,300,540 | 12.50 Gb | 97.71 | 93.51 | 81.22% |
| **Average** | 95,795,386 | 14.37 Gb | 91,617,924 | 13.75 Gb | 97.42 | 93.02 | 78.07% |
| **Total** | 574,772,318 | 86.20 Gb | 549,707,542 | 82.52 Gb |  |  |  |
